# Perceived health risks from paid work in Germany in 2014/2015 and 2024

**DOI:** 10.25646/13635

**Published:** 2026-02-04

**Authors:** Florian Beese, Stephan Müters, Ronny Kuhnert, Nico Dragano, Jens Hoebel

**Affiliations:** 1 Robert Koch Institute, Department of Epidemiology and Health Monitoring, Berlin, Germany; 2 Institute of Medical Sociology, Centre for Health and Society, Medical Faculty and University Hospital, Heinrich Heine University Düsseldorf, Düsseldorf, Germany

**Keywords:** Working Conditions, Employment, Health Status, Prevalence, Health Promotion, Health Inequality, Germany

## Abstract

**Background:**

Paid work can have a significant impact on employees’ health. This article describes the perceived health risks associated with paid work in Germany.

**Methods:**

Data from full-time and part-time employed persons aged 18 to 64 from the GEDA 2014/2015-EHIS study (6,782 women; 6,170 men) and the 2024 ‘Health in Germany’ panel (10,634 women; 8,907 men) were analysed. The perceived health risk posed by paid work was measured using a four-point Likert scale and evaluated on an age-standardised basis by gender and level of qualification.

**Results:**

Approximately one quarter of the working population reported a high or very high health risk from their paid work in both survey periods. Among women, the prevalence in 2024 was higher than around ten years earlier. The highest prevalence was found among men with a low level of qualification.

**Conclusions:**

Working conditions remain key entry points for prevention and health promotion – also with regard to health equity.

## 1. Introduction

Paid work can be both beneficial and stressful for physical and mental health [1, 2]. However, work can also contribute to a sense of purpose, social well-being and financial security [3]. At the same time, globalisation, digitalisation and demographic change are profoundly altering the world of work, creating new opportunities but also leading to new types of stress and health risks, such as the blurring of boundaries between work and private life due to constant availability [4] or stress caused by the use of digital technologies such as computers, smartphones, emails, etc. (tech-nostress) [5]. Stress in the workplace – for example, due to physically demanding activities, shift work or psychosocial stress – is associated with an increased risk of illness [6–8]. In addition, there are considerable social inequalities in access to healthy working conditions: people with lower levels of qualification are more likely to work in occupations with higher health risks [6, 9]. Consequently, paid work influences both the maintenance of health and the development of diseases and opens up opportunities for preventive measures – also in the context of health inequalities.

This article reports on a general indicator that reflects the perceived health risk posed by one's current paid work. The indicator has already been collected several times in health surveys conducted by the Robert Koch Institute (RKI). Previous results showed that 16.8 % of respondents in 2010, 20.9 % in 2012 and 23.0 % in 2014 perceived a high or very high health risk from paid work [10–12]. Furthermore, the results indicate that the indicator is associated with both physical (e.g. lifting/carrying heavy loads) and psychosocial stressors (e.g. working under time/performance pressure) [10]. When comparing the results across several surveys, it should be noted that the study design (sampling, survey mode) differed between the 2010 and 2012 studies and the 2014 study. These differences can produce systematic mode and selection effects, meaning that observed changes cannot be clearly separated from design-related influences. This article compares the results of perceived health risks from paid work from two methodologically comparable surveys from 2014/2015 and 2024.

**Key messages**

► Overall, approximately one quarter of the working population in both 2014/2015 and 2024 perceive their work as posing a high or very high risk to their health.
► Men with a low level of qualification report perceived health risks from their work more frequently in both survey periods.
► In 2024, there is a higher prevalence of perceived health risks from paid work among women compared to 2014/2015.

## 2. Methods

The data is based on the studies German Health Update 2014/2015 (GEDA 2014/2015-EHIS; November 2014 – July 2015) and the 2024 annual survey of the RKI's ‘Health in Germany’ panel (RKI Panel 2024; May 2024 – January 2025; data set version 5). In both surveys, the indicator ‘Perceived health risk from paid work’ was collected by asking employed persons ‘Do you believe that your health is at risk from your work?’ The response categories were ‘very high’, ‘high’, ‘moderate’ and ‘not at all’. For this article, the answers were dichotomised so that a high to very high perceived health risk from paid work is shown in contrast to a moderate to no perceived health risk.

The analysis was limited to individuals aged 18 to 64 who were employed full-time or part-time. Gender was surveyed in GEDA 2014/2015-EHIS with the question: ‘Are you male or female?’ In the RKI Panel 2024, both the sex assigned at birth and gender identity (including open-ended response options) were recorded. Consequently, gender identity was used in this analysis. Gender-diverse individuals were included in the sample description but were excluded from further analysis due to statistical uncertainties resulting from the small number of cases (n = 40; Annex Table 1), they were excluded from further analysis. In addition to gender and age, the formal level of educational and professional qualifications was included in the analyses. This was collected using the CASMIN classification (Comparative Analysis of Social Mobility in Industrial Nations) and divided into the categories low (primary to lower secondary education), medium (medium/higher secondary education) and high (tertiary education) [13].

**RKI-Panel ‘Health in Germany’ 2024**

**Data holder:** Robert Koch Institute

**Objectives:** To provide comprehensive data on the health status, health-related behaviour and health care of the population in Germany, with the future possibility of longitudinal comparisons and analysis of trends over time

**Study design:** Panel study with a mixed-mode approach (online and written-postal participation)

**Population:** German-speaking population aged 18 and over in private households with main residence in Germany

**Sample:** Probabilistic/representative sample of the Health in Germany panel infrastructure

**Participants in the 2024 annual wave:** A total of 41,376 of the persons registered in the panel took part in at least one of the three sub-waves in 2024.

Questionnaire A: 14,759 women, 12,374 men,

66 persons with other gender identities

Questionnaire B: 14,828 women, 12,258 men,

61 persons with other gender identities

Questionnaire C: 14,709 women, 12,329 men,

64 persons with other gender identities

Questionnaire D: 14,872 women, 12,368 men,

66 persons with other gender identities

**Data collection:**

1st sub-wave: 28.05.2024 to 05.08.2024

2nd sub-wave: 12.08.2024 to 14.10.2024

3rd sub-wave: 28.10.2024 to 06.01.2025

More information at www.rki.de/panel-en

Observed and age-standardised (based on the 2013 European standard population [14]) prevalences of perceived health risks associated with paid work by gender and level of qualification are reported. For model-based control of employment status (full-time or part-time employment), Pois-son regressions [15] were additionally estimated to calculate predicted probabilities for each survey period and for women and men respectively, which are reported stratified by level of qualification. The predicted probabilities allow the prevalences for both survey periods to be compared independently of age and employment structure.

**GEDA 2014/2015-EHIS**

**Data holder:** Robert Koch Institute

**Objectives:** To provide reliable information on the population’s health status, health-related behaviour and healthcare in Germany, with the possibility of a European comparison

**Method:** Questionnaires completed on paper or online

**Population:** People aged de years and above with permanent residency in Germany

**Sampling:** Registry office sample; randomly selected individuals from 301 municipalities in Germany were invited to participate

**Participants:** 24,016 people

(13,144 women and 10,872 men)

**Data collection:** November 2014 – July 2015

More information at www.rki.de/geda

Deviations of the samples from the population structure were corrected using weighted calculations with regard to gender, age, federal state and education, as well as in GEDA 2014/2015-EHIS with regard to district type and in the RKI Panel 2024 with regard to municipality size class and household size. In addition, the sample points were taken into account for the calculation of robust standard errors. Detailed descriptions of the study designs and weightings can be found in Lange et al. (2017) for GEDA 2014/2015-EHIS [16] and in Lemcke et al. (2024) for the RKI Panel 2024 [17].

## 3. Results

A total of 12,952 people aged between 18 and 64 who were in full-time or part-time employment were included in the analyses for GEDA 2014/2015-EHIS and a total of 19,541 people for the RKI Panel 2024 (Annex Table 1). In 2014/2015, the observed prevalence of a perceived high or very high health risk from paid work in this population group was 24.5 % (women 20.5 %, men 27.8 %), while in 2024 it was 25.3 % (women 23.6 %, men 26.9 %).

Even after age-standardisation, the overall prevalence is lower among women than among men, with the prevalence among women being lower in 2014/2015 than in 2024 (Table 1). Furthermore, no age differences were observed among women in 2014/2015, whereas in 2024 the prevalence decreased with increasing age. Among men, there were no significant differences between age groups, but there were differences according to the level of qualification. Among men with a low level of qualifications, the prevalence was about twice as high as among men with a high level of qualifications. Among women, there were only minor differences in terms of the level of qualification.

**Figure 1:**
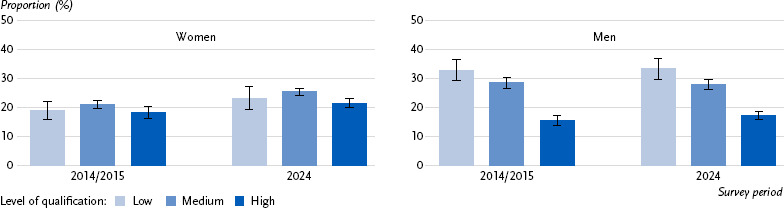
Age-standardised prevalence of perceived health risks from paid work by gender and level of qualification, adjusted for employment status, 2014/2015 and 2024. Source: GEDA 2014/2015-EHIS (n = 6,782 women; n = 6,170 men), RKI Panel 2024 (n = 10,634 women; n = 8,907 men)

Even after additional adjustment for employment status (full-time, part-time), there are no significant changes in the prevalences by levels of qualification when comparing the two survey periods. While marginal differences by levels of qualification can be seen among women, the gradient among men remains the same for both survey periods, with particular differences between men with a low/medium level of qualification compared to men with a high level of qualification (Figure 1, Annex Table 2)

## 4. Discussion

Overall, approximately one quarter of full-time or part-time employed persons in Germany perceive their employment as posing a high or very high risk to their health. Men with low or medium levels of qualification in particular show higher prevalences in both survey periods compared to men with high levels of qualification. Among women, there was a slight increase in prevalence over time, as well as a higher prevalence among young and middle-aged employed persons in 2024.

Between 2014 and 2024, the employment rate rose from 69.3 % to 74.1 %, particularly among women [18]. At the same time, women performed 44.3 % more unpaid (care) work than men in 2022 [19]. Women in younger and middle age groups in particular may be more likely to experience multiple burdens due to labour force participation and parallel care work. At the same time, social debates about equality, work-life balance and role models [20] may contribute to a more sensitive perception of work-related burdens that may previously have been normalised. Recent findings also show that structural changes such as flexible working time models do not automatically provide relief: for women, the relieving effect depends heavily on the distribution of housework and care work; if this remains unevenly distributed, flexibility can even lead to greater work-life conflicts [21]. Against this background, multiple burdens and compatibility conflicts could play a role in the perception of health risks associated with paid work, even if the indicator is specifically queried in the context of paid work. In addition, the results could also be interpreted in the context of the COVID-19 pandemic: women are more likely to be employed in professions that involve interactive work (e.g. health and education professions). These occupations were often associated with higher health risks in the context of the COVID-19 pandemic [22], while at the same time being associated with a lack of recognition, high physical and psychological workloads and comparatively low pay [23]. These factors may have contributed to an increased perception of health-threatening working conditions, particularly in these occupations.

With regard to the level of qualification of respondents, stable inequalities over time are particularly evident among men. Occupations that more often require a low level of qualification [6] are also more often associated with physical (e.g. lifting heavy loads) and psychosocial (e.g. limited scope for action and decision-making, precarious employment) workloads, e.g. in civil engineering, (interior) construction or building services engineering. These occupations are largely performed by men [24, 25]. The associated strains are correspondingly common among men and could explain the continuing inequalities among men.

This indicator has already been used in previous studies as a summary measure of perceived health-related workloads. This allows health reporting to take into account employees' perspectives on their working conditions. However, perceived health risks can be influenced by individual health concepts, expectations, experiences and social discourse, and therefore do not directly reflect objective working conditions.

Work remains a key setting for prevention and health promotion. In addition to occupational safety and health management, measures that take into account different working conditions and stresses are becoming increasingly important – such as reducing physical and psychosocial risks in physically demanding jobs or promoting working time models that improve the work-life balance. Groups with structurally increased occupational risks or multiple stresses could benefit particularly from this. Targeted improvement of working conditions, especially in occupations with lower formal qualification requirements, can also contribute to reducing social and health inequalities.

## Figures and Tables

**Table 1:** Prevalence of perceived health risks from paid work by gender, age and level of qualification. Source: GEDA 2014/2015-EHIS (n = 6,782 women; n = 6,170 men); RKI Panel 2024 (n = 10,634 women; n = 8,907 men)

	2014/2015				2024			
	Women		Men		Women		Men	
	%	(95 % CI)	%	(95 % CI)	%	(95 % CI)	%	(95 % CI)
**Age (years)**								
18 – 29	23.7	(20.5 – 26.9)	26.0	(22.2 – 29.8)	29.1	(25.7 – 32.6)	26.5	(23.1 – 29.9)
30 – 44	18.7	(16.7 – 20.6)	28.4	(25.9 – 30.9)	23.7	(21.9 – 25.4)	28.5	(26.3 – 30.8)
45 – 64	20.4	(18.7 – 22.1)	28.0	(26.1 – 29.8)	21.8	(20.4 – 23.2)	25.7	(23.9 – 27.4)
**Level of qualification (CASMIN)** ^1^								
Low	18.8	(15.1 – 22.5)	33.1	(29.1 – 37.2)	23.8	(18.4 – 29.2)	33.5	(29.0 – 38.1)
Medium	21.2	(19.6 – 22.8)	29.1	(27.1 – 31.0)	25.4	(23.9 – 26.8)	28.2	(26.4 – 30.0)
High	19.5	(16.6 – 22.4)	14.1	(12.0 – 16.1)	22.3	(20.0 – 24.5)	16.8	(15.1 – 18.6)
**Total** ^1^	**20.3**	**(19.0 – 21.6)**	**27.0**	**(25.5 – 28.5)**	**24.0**	**(22.8 – 25.3)**	**26.9**	**(25.5 – 28.3)**

^1^ age-standardised prevalence (European standard population 2013); weighted % and 95 % CI

CI = confidence interval, CASMIN = Comparative Analysis of Social Mobility in Industrial Nations

**Annex Table 1:** Sample composition. Source: GEDA 2014/2015-EHIS, RKI Panel 2024

	GEDA 2014/2015-EHIS (N = 12,952)	RKI-Panel 2024 (N = 19,581)
	n (%)^a^	n (%)^a^
**Gender**		
Women	6,782 (44.6)	10,634 (47.0)
Men	6,170 (55.4)	8,907 (52.8)
Gender-diverse	–	40 (0.2)
**Age (years)**		
18 – 29	1,963 (17.8)	2,916 (16.0)
30 – 44	4,330 (33.2)	6,834 (36.3)
45 – 64	6,659 (49.0)	9,831 (47.7)
**Level of qualification (CASMIN)**		
Low	1,723 (19.9)	1,879 (22.5)
Medium	7,629 (60.8)	10,286 (52.7)
High	3,595 (19.3)	7,410 (24.8)
Missing	5	6
**Employment status**		
Full-time	9,786 (78.2)	13,613 (72.2)
Part-time	3,166 (21.8)	5,968 (27.8)
**High/very high perceived health risks associated with paid work**	3,000 (24.5)	4,599 (25.3)
Missing	153	209

^a^n unweighted; % weighted; CASMIN = Comparative Analysis of Social Mobility in Industrial Nations

**Annex Table 2:** Age-standardised prevalence of perceived health risks from paid work by gender and level of qualification, adjusted for employment status, 2014/2015 and 2024. Source: GEDA 2014/2015-EHIS (n = 6,782 women; n = 6,170 men); RKI Panel 2024 (n = 10,634 women; n = 8,907 men)

Level of qualification (CASMIN)	2014/2015				2024			
	Women		Men		Women		Men	
	%	(95 % CI)	%	(95 % CI)	%	(95 % CI)	%	(95 % CI)
Low	19.0	(15.6 – 22.4)	33.0	(29.2 – 36.8)	23.2	(19.0 – 27.5)	33.7	(29.7 – 37.7)
Medium	21.1	(19.5 – 22.7)	28.6	(26.7 – 30.5)	25.2	(23.7 – 26.7)	28.3	(26.4 – 30.1)
High	18.3	(16.0 – 20.5)	15.6	(13.8 – 17.5)	21.6	(20.0 – 23.3)	17.3	(15.8 – 18.7)

CI = confidence interval; CASMIN = Comparative Analysis of Social Mobility in Industrial Nations
